# Poisoning by Paraldehyde

**Published:** 1900-09-29

**Authors:** 


### POISONING BY PARALDEHYDE.

A fatal case of poisoning by paraldehyde is reported by Dr. Lovell Drage,[1] a case which, while on the one hand showing that this drug must always be administered with care, shows on the other hand how large a dose may be required to produce its lethal effect. The patient in this case was 46 years of age, and had suffered for some years from chronic emphysema, bronchitis, and heart disease, and had been in the habit of taking drugs of various kinds to excess. The amount of paraldehyde ordered was two teaspoonfuls in a wineglassful of water thrice during the night. However, in the course of the 24 hours, roughly speaking, ten drachms of the drug were used, and in the next nine hours three drachms. Dr. Drage seems to think that even more than this was really given. The symptoms noted by the nurse were restlessness and some degree of delirium and constant craving for the drug. About two hours after the nurse had given the last dose the patient possessed herself of another bottle containing at least six ounces, and out of this, according to the estimate made by the nurse, she consumed about two ounces. The condition of the patient then became so serious that a medical man was sent for, and on his arrival he found the following symptoms: Unconsciousness, profuse perspiration, rather deep cyanosis, intermittent pulse and shallow breathing, about normal in time. Death occurred about three hours after the lethal dose was taken. Altogether it would seem that 13 drachms of the drug were administered in the course of 33 hours before the patient herself took the fatal dose, which Dr. Drage says was possibly less than the amount stated. According to the German Pharmacopœia 85 drops are the maximum single dose, and 170 drops the maximum quantity to be given in the 24 hours, and Dr. Drage, speaking from a considerable experience in the use of the drug, regards these doses as correct.

[1] Lancet, Sept. 22.

